# Im/Mobile Boundaries of Intimacies: gender, family and mobilities in contemporary urban India

**DOI:** 10.3389/fsoc.2026.1857536

**Published:** 2026-10-23

**Authors:** Vaishnavi Mahurkar, Mayurakshi Chaudhuri

**Affiliations:** Flame University, Pune, India

**Keywords:** boundary work, family, gender, intimacies, mobilities

## Abstract

The widespread integration of digital technologies in India since the 2000s has reshaped social identities and mobilities, particularly redefining relationship boundaries. In this context, this article examines how boundaries of intimacies are negotiated across digital and physical spaces in contemporary urban India. Drawing on primary data collected between 2023 and 2024 and utilizing Grounded Theory methodology, this article illustrates how social, political, economic and the technological forces renegotiate intimacies and social mobilities. We bring together two key areas of scholarship that have hitherto contributed independently to an informed understanding of social mobility and its negotiations: the New Mobilities paradigm and the Boundary Work framework, and we illustrate a novel analytical framework particularly to capture the *simultaneity of negotiations* within various forms of intimacies. We term this novel framework “*Im/Mobile Boundaries of Intimacies,”* which builds on the debates around mobilities, gender, and intimacies and extends scholarly understandings of how contemporary intimate lifestyles are restructured within and beyond long-standing social institutions such as family and marriage. The study contributes towards an analysis of imaginative and mobilities practices that young adults engage in order to forge meaningful intimacies in contemporary urban contexts.

## Introduction

Family and marriage as enduring social institutions have long structured intimate life and organisations globally (Rubin, 2002). This discussion has included normative kinship frameworks (Karve, 1953; Das, 1976; Uberoi, 1993), emerging transformations in gender and sexuality norms (Vanita and Kidwai, 2000; Narrain and Bhan, 2005), and contestations around meaning of family and marriage (Chowdhry, 2007; Sinha, 2022; Bhandari, 2024), with ongoing marriage equality debates (Sheikh and Samuel, 2024; Supriyo v. Union of India, 2023). The widespread integration of digital technologies in India since the 2000s has reshaped social identities and social mobilities, particularly redefining relationship boundaries which have only complicated these ongoing debates.

One key relationship boundary negotiation has been that of intimacies.^1^ In the literature and in practice, intimacies are often conceptualised as increasingly private or individualised (Giddens, 1992). However, intimacies are deeply embedded within historically specific social, cultural and political contexts. Scholarship on intimacies in India has predominantly examined such negotiations through the lens of cisgender, heterosexual endogamous marriages (Sen et al., 2011; Uberoi, 2006; Mody, 2008). While heteronormative marriage practices continue to exert influence in the current Indian social landscape, such practices and their interpretations often limit the understanding of contemporary intimate practices in urban India. Although recent scholarship has discussed pre-marital relationships, this literature presupposes marriage as ultimate goal (e.g., Bhandari, 2017). As a result, intimacies that lie outside normative marriage frameworks are often either understood as marginalised or remain underexamined. Addressing this lacuna in scholarship and in public social understanding require close examination of the processes which shape both possibilities and constraints of intimate life. This becomes even more essential particularly as people move across geographical spaces, time and digital platforms. Such examinations raises questions on how young adults negotiate intimacies in a context where digital technologies such as access to personal mobile phone, social media and dating applications (henceforth as ‘apps’) increasingly reshape relational life, even as enduring social structures of heteronormative marriage continue to exert influence.

Drawing on primary data collected between 2023 and 2024 and utilizing Grounded Theory methodology (Strauss and Corbin, 1998), this article illustrates how social, political, economic and technological forces renegotiate intimacies and social mobilities. This study brings together voices across 30 semi-structured in-depth interviews across the Indian metropolises of Bengaluru, Mumbai, and Pune. The study aimed to capture a wide range of ways in which young adults in India negotiate intimacies, and how they attach meanings of social mobility to their social locations. We maximized the sample in terms of people’s social attributes, contexts and experiences. In other words, we include a multiplicity of views corresponding to people’s diverse “social location[s]” (Mahler and Pessar, 2001, p. 445) in our analysis, while bringing in significant voice(s) from key informants.

The study contributes towards an analysis of imaginative and mobilities practices that young adults engage in order to forge meaningful intimacies in contemporary urban contexts. To illustrate, we bring together two key areas of scholarship that have hitherto contributed independently to informed understanding of social mobility and their negotiations: the New Mobilities paradigm (Sheller and Urry, 2006), and the Boundary Work framework (Lamont and Molnár, 2002). Integrating these two key scholarly avenues enables a novel analytical framework designed to capture the *simultaneity of negotiations* within various forms of intimacies. We term this novel framework as “Im/Mobile Boundaries of Intimacies”. This framework builds on the debates around mobilities, gender, and intimacies and extends scholarly understandings of how contemporary intimate lifestyles are restructured within and beyond long-standing social institutions such as family and marriage. A vignette drawn from our data illustrates this restructuring of intimate lifestyles. The article then reviews key literature on gender and intimacy, the New Mobilities paradigm, and the Boundary Work framework; outlines the methodological approach; applies the novel analytical framework to examine the data; and concludes with contributions and future research directions.

### Voicing Shalini

Shalini is a twenty-nine-year-old, cisgender, heterosexual woman of Tamil Brahmin heritage. Interviewed in September 2023 while residing in Pune, Shalini reflected on her varied geographic experiences. She has lived in multiple Indian metropolises such as in Mumbai and Bengaluru as well as in rural areas. Professionally engaged in the social services sector, she is also a research scholar with aspirations for doctoral studies. Growing up, though Shalini’s friends found ‘boys’ attractive, Shalini always felt that they [the ‘opposite gender’, i.e., boys/men] were her competitors.^2^

Looking back at her early experiences and her recent relationship negotiations, Shalini reflected that perhaps her persistent desire for intellectual compatibility steered her away from various potential connections. According to Shalini, “My constant quest to discover someone whose intellect resonates with mine, I believe, has served as a barrier, deterring me from various experiences. That’s my perspective on the matter.”

Shalini’s move from Mumbai to Bengaluru for her Master’s degree marked a shift from living with family in Mumbai to an independent urban living situation. Shalini described that she underwent notable transformation becoming more extroverted, unafraid of confrontations. Paradoxically, despite this newfound assertiveness, Shalini noted continued hesitation in initiating romantic interactions.

It was during this phase that Shalini was exposed to the ‘culture’ of dating apps. Shalini observed her roommate’s engagement with dating apps, introducing her to a previously unexplored domain. In fact, Shalini shared how she never believed she would need to use a dating app before she moved to Bengaluru. Shalini explained, “And I thought, Oh my god, this is a big way in which people date! But *tabhi bhi mujhe lagta tha ki pata nai shayad mujhe jarurat padegi ya nai padegi.* [Even then, I still thought I might not need the dating apps.].” She adds, “I had this notion that someone would simply materialize, sparing me from the effort. I was apprehensive about investing the effort because, let’s face it, it’s intimidating! Putting yourself out there, searching for someone, engaging in conversations, figuring it all out—it felt like a daunting task.” While this move opened up new possibilities, Shalini characterized the process of “putting oneself out there” as “intimidating.”

Acquiring new perspectives on dating prompted Shalini to rethink and even question traditional romantic practices. However, Shalini’s reluctance to date (online or in person) was influenced by her family’s faith in Hindu astrology. This belief in predetermined marital timelines fundamentally influenced Shalini’s perspective on the necessity of dating and diminished her need to date.

Shalini’s experiences are far from unique; they exemplify broader patterns in our dataset. Her experiences illustrate how social mobilities and boundary-settings shape the negotiation of intimacies. In the following section, we turn to discussing briefly three key and already-expansive bodies of social science literature that inform this research: (1) Gender, Family and Intimacies; (2) the New Mobilities Paradigm and (3) the Boundary Work framework, enroute to examining the patterns in our data.

#### Gender, family and intimacies: conceptual foundations

Feminist and queer scholarship have been foundational to understanding the evolving meanings of family, gender, sexuality and intimacies. This literature demonstrates that gender and sexuality are key sites through which intimacies are contested and policed particularly in relation to family, marriage, respectability and social reproduction (Rubin, 2002; Puri, 1999; Beasley and Holmes, 2016). Intimate relations are deeply structured by gender and sexuality norms, power asymmetries and the moral fabric of a given society shaping who can desire, who is deemed desirable, and how social status and hierarchy are negotiated within both private and public spheres. While notions of sexuality and intimacies are often used interchangeably, scholars argue that sexuality extends beyond sexual acts and desires and encompasses institutions, identities, social assumptions, and customs, as well as resources and labour (Beasley and Holmes, 2016; Jackson, 1999). In contrast, scholarship on intimacies draws attention to broader emotional, relational, embodied connections that extends and includes care, attachment, and forms of closeness. This literature emphasises that intimate life is shaped by the social expectations, moral norms and relational obligations that extend beyond the dyad (Roseneil and Budgeon, 2004; Jamieson, 2005; Budgeon, 2008; Smart, 2007). Viewed through an intersectionality lens, feminist and social constructionist approaches further demonstrate that gender, sexuality and intimacies are co-constituted through intersecting power geometries - of caste, class, and religion. Rather than operating independently, these axes of social differentiations shape legitimate, respectable, or acceptable intimate relations (Jackson, 1999; Weeks, 2002; Menon, 2012).

Across many global contexts including in South Asia, the biological condition of the body (i.e., the sex of the body) is often coterminous with the idea of a gender binary (male/female inevitably translating into man/woman) and rigidly enforced gender norms (Shah et al., 2015). Sexual interactions are restricted to the gender binary, defining monogamous, heteronormative marriage for reproductive purposes (Narrain and Bhan, 2005; Menon, 2012). Postcolonial feminist scholarship further highlights the continuing link between sexuality and colonialism. Puri (1999), for instance, shows how privileged heterosexual urban women in India are aware of and negotiate national sexual norms, that position sexual intimacy as ‘properly’ belonging within marriage, even as they resist these norms in a variety of ways. Recent research such as Lamba (2022), for example, illustrates how young urban middle class women exercise greater choice in intimate and marital relationships while continuing to navigate familial expectations and wider caste and gender norms. This raises questions about what constitutes legitimate and acceptable intimacies in contemporary India, and by extension, in South Asia. Gender and sexuality emerge as critical sites of boundary construction intertwined with respectability politics and the intersections of caste, religion, class, age, and ableism. Simultaneously, these fluid boundaries are constantly negotiated, transgressed, and shifted providing fertile ground for examining how boundary work reproduces and diffuses longstanding norms (Stein, 1997). This in turn redefines questions of mobility and immobility in family, marriage, and society.

#### The new mobilities paradigm

The social sciences and the humanities disciplines experienced a “mobility turn” in the early 2000s giving rise to the “New Mobilities” paradigm (see Hannam et al., 2006; Sheller and Urry, 2006; Urry, 2007). This scholarship challenged the ‘sedentarist’ tendency in social sciences, to treat social life, institutions and identities as territorially fixed, stable and rooted in place. This paradigm also called for different forms of movements, including change of geographical location such as migration, to be analysed and understood comparatively to other forms of movements associated with globalisation, such as flows of massive capital and goods (Salazar, 2017). Mobilities scholars emphasised on movement and flow as constitutive of social relations, identities and social institutions (Sheller and Urry, 2006; Cresswell, 2006).

In this literature, scholars argue that mobility serves as a metaphorical representation of the contemporary world, encompassing both its tangible manifestation and its conceptual implications (Salazar, 2011, 2017; Urry, 2007). Mobility emphasizes movement and process above stability in both space and time. From this perspective, mobilities are not simply outcomes of social processes or contextual backdrops, but productive forces through which social relations such as gender, identities and even social institutions such as family and marriage are continually remade. Although people are perpetually in motion, not all movements hold the same significance and capacity to transform lives. Furthermore, movements can be uneven and stratified and shaped by power, access and social positioning (Salazar, 2010; Salazar and Smart, 2011; Glick Schiller and Salazar, 2013; Chaudhuri and Thimm, 2018).

India’s emerging “new middle class” - professionals particularly those migrating intra-nationally for employment and higher education - exemplifies novel forms of socio-economic mobility (Bhandari, 2020; Upadhya, 2009, 2011; Shukla and Chaudhuri, 2023; Brosius, 2012; Fernandes, 2011). This mobility is interwoven with sociality, family, and marriage patterns and shaped by intersecting relations of class, caste and other forms of difference (Chaudhuri et al., 2024; Mahler et al., 2015; Divya et al., 2010; Upadhya, 2004). At the same time, mobile and digital technologies create new possibilities for negotiating intimate and familial relationships, while also reproducing and challenging gendered relations and domestic boundaries (Donner et al., 2013; Doron, 2012) The mobilities lens therefore offers a way to examine how intimacies are produced, negotiated, and transformed through movements, flows and differential access to forms of mobility. It also draws attention to how gender, sexuality, class, caste and underlying power relations shape the possibilities of reproducing or transgressing social norms through everyday mobility practices.

More recent iterations of the mobilities paradigm have extended attention to digitally mediated forms of mobility, as mobile phones, social media and online platforms reshape how people communicate, sustain relationships and negotiate identities. In India, mobile communication intersects with courtship, marriage and kinship, simultaneously reinforcing and challenging existing gender dynamics and authority structures (Doron, 2012). Recent research on digital intimacy in Asia demonstrates that while digital mediation creates new avenues for negotiating family, marriage and intimate relationships, these new possibilities for intimacies remain entrenched within existing structures of gender, caste, class and social expectations (Niranjana and Lee, 2022; Niranjana and Lukose, 2026). This broader transformation is also visible in Indian media and industry reports indicating increase in use of online dating platforms during and following the COVID-19 pandemic, including growing uptake in Tier I and Tier II cities (Hariharan, 2021; Jha, 2022). Such developments invite closer attention to how digitally mediated everyday practices reshape intimate and familial relations, while also creating new sites for negotiating gendered and social boundaries. Further they also reveal the uneven ways in which access to and use of digital technologies are experienced across social locations.

At its core, mobility is characterized by *boundary-crossing* (Salazar, 2018). This insight is particularly relevant to the present study which examines mobility in relation to gender, family and intimacies. Bringing mobility in conversation with boundaries draws attention to how boundaries around intimacies may be negotiated, maintained, transgressed or loosened. We therefore now turn to scholarship on boundaries, and the Boundary Work framework in the next section to advance this discussion further.

#### Boundary work and intimacies

The Boundary Work framework developed originally through research on race, ethnicity and migration, particularly in the North American context, and expanded across a range of social and institutional contexts globally (Barth, 1969; Lamont and Molnár, 2002; Wimmer, 2008). Lamont and Molnár’s (2002) review of scholarly work on boundaries and boundary work suggests that the concept of boundaries has been associated with cognition, social and collective identity, comparability, census categories, cultural capital, cultural membership, hegemonic masculinity, and professional jurisdictions, to name a few. Lamont and Molnár’s (2002) review highlights the value of boundaries for examining relational processes across different social locations and institutions. In their study, Lamont and Molnár (2002) distinguish between two types of boundaries: “symbolic” and “social.” They argue that while symbolic boundaries are “conceptual distinctions made by social actors to categorize objects, people, practices, and even time and space”, social boundaries “are objectified forms of social differences manifested in unequal access to and distribution of resources (material and nonmaterial) and social opportunities” (Lamont and Molnár, 2002, p. 168). This distinction is useful for understanding how everyday processes of categorisation and evaluation can be consequential for social relations, producing inclusion, exclusion and differentiation.

For scholars studying intimacies, the Boundary Work framework is useful in examining how people establish, maintain, and blur boundaries in order to develop and negotiate intimacies both within and outside of normative institutions. Alba (2005) distinguishes between “bright” boundaries, where the distinctions are relatively clear and unambiguous, and “blurry” boundaries, where individuals may occupy more ambiguous positions. Alba (2005) further emphasises that these distinctions result in varying permeability of boundaries and that boundaries are constructed from cultural, legal and institutional material already at hand. Additionally, these boundaries, Favell (1998) contends, depend in a path-dependent way on the prior histories of the societies and groups involved. Ultimately, they are sociologically complex, taking different forms and carrying myriad meanings across social contexts.

The above context opens up two critical avenues through which intimacies become sites of boundary work: *constructions* of boundaries, and their *permeability* (Bryson, 1996; Erickson, 1996; and Peterson and Kern, 1996). French sociologist Pierre Bourdieu’s contributions to comprehending the constructions and permeability of social boundaries have wielded significant influence within this scholarly discourse. Subsequent work has advocated for a more nuanced perspective on symbolic and social boundaries, which urged for an exploration of the permeability and varying significance of diverse boundary forms across different contexts such class, race/ethnicity, gender/sexuality, and employment (Lamont, 1992; Lareau, 2000; Jackson, 1999; Young, 2004). More recent research delves into the relational character of boundary making, highlighting how social identities and distinctions are produced through multiple intersecting relations, transcending simplistic binary frameworks.

##### Intimacies as boundary work

One strand of scholarship on intimacies examines how boundaries and boundary work can be employed to theorize intimacies (Jamieson, 2005; Roseneil, 2005). Scholars argue that the process of forming intimate relationships and maintaining them involves complex processes of social and moral boundary crossing. Intimacies is frequently understood through a specific sort of “closeness,” familiarity, privileged knowledge, deep emotional ties, trust and being “special” to another person. Yet, as Jamieson (2005) argues, the production and maintenance of intimacies also involves boundary work: boundaries may be lowered among intimates while being heightened between intimates and those positioned outside the intimate relationship. In accounts of personal life, intimates are described as if encapsulated together by a protective boundary that stops distractions that would otherwise interfere with their intimacies or by an exclusionary boundary that keeps non-intimates out. This understanding of intimacies as involving both inclusion and exclusion highlight the active boundary work involved in production and negotiation of intimate boundaries. Research on queer intimacies in India shows how caste can shape the negotiation of desire and interaction, including in digitally mediated spaces (Borisa, 2020). Recent scholarship on digital intimacy further highlights the processes of boundary-drawing and the negotiation of intimate connections across online and offline contexts (Niranjana and Lee, 2022). Thus, the Boundary Work framework is helpful in comprehending negotiation of power and agency in family, marriage, and beyond in forging and maintaining intimacies. This discussion further highlights that gender and sexuality, while crucial, are not the standalone analytical categories but are inflected by relational dimensions of caste and class.

The New Mobilities paradigm outlined in the earlier section thus provides a critical lens to look at boundary work in the construction and negotiation of intimacies through time and complex places and spaces, including the digital. By bringing these two well-established areas of scholarship, i.e., New Mobilities and Boundary Work, into conversation, we anticipate more effective understanding of shifts in both self-ascribed and attributed meanings to intimacies (Frello, 2008). The movement, then, is not restricted to physical motion only, but extends to flows of ideas, meanings and constructions of intimacies *simultaneously*.

#### Methodology, methods, and data

This study employs Grounded Theory (Strauss and Corbin, 1998), particularly Constructivist Grounded Theory (Charmaz, 2017) as the primary research methodology, allowing data collection and analysis to inform each other concurrently.^3^ Based on the principle of “theoretical sampling,” new data sources were constantly sought during data collection phases to maximize conceptual variation based on emerging insights. The research is grounded in an ethnographic approach, comprising 30 in-depth, semi-structured interviews conducted between early 2023 and early 2024 across the Indian metropolises of Bengaluru, Mumbai and Pune. The interviews were in-depth and semi-structured, and discussions allowed for probes to evolve dynamically in response to newly identified themes. This iterative qualitative approach ensured a comprehensive understanding of the participants’ experiences.

The following overarching questions have structured this research: (1) How are symbolic and social boundaries around intimacies constructed, negotiated, and reified amid emerging transformations in relationships in contemporary India?; and (2) How do changing digital and virtual spaces influence the construction, negotiation and reconfiguration of social and symbolic boundaries of intimacies?

A multiple snowball sampling technique was employed to recruit informants in this study. Specifically, two sampling strategies were adopted: (a) Convenience Sample; and (b) Theoretical Sampling. Convenience samples, or “availability” samples (Babbie, 1989; Mutchnick and Berg, 1996), rely on available subjects, that is, those who are close at hand or are otherwise available. Under certain circumstances like in this case where the population is unknown, random sampling was not possible and therefore convenience sampling proved to be an excellent means of obtaining valuable and reliable information (Berg, 2007). Convenience sampling also enabled inclusion of heterogeneity in the dataset.

Another lead for being attentive to sample heterogeneity in this study can be found in what Marcus (1995) described as “multi-sited ethnography” (Marcus, 1995, p. 95) and its benefits to maximize heterogeneity in research (Marcus, 1995, p. 102),

“In projects of multi-sited ethnographic research, de facto comparative dimensions develop instead of a function of the fractured, discontinuous plane of movement and discovery among sites as one maps an object of study and needs to posit logics of relationship, translation, and association among these sites. Thus, in multi-sited ethnography, comparison emerges from putting questions to an emergent object of study whose contours, sites, and relationships are not known beforehand, but are themselves a contribution of making an account that has different, complexly connected real-world sites of investigation. The object of study is ultimately mobile and multiply situated, so any ethnography of such an object will have a comparative dimension that is integral to it, in the form of juxtapositions of phenomena that conventionally have appeared to be (or conceptually have been kept) ‘worlds apart.’”

Following this principle of establishing a comparative relationship between the social agents and associated social practices they perform, multi-sited ethnography was conducted for this study focusing particularly on the three metropolises discussed above.

### Sample representativeness, choosing a representational voice, and arriving at a collective story

A natural corollary that follows sampling is the representativeness of the data. The central question in “telling the story” then becomes: whose voice(s) and experiences are chosen to narrate the story? Richardson (1990, p. 39) cautions qualitative researchers that,

“Deciding how to present voices and lives is a continuous problem for qualitative writers. Because we use the voices and experiences of the people we study, both for their own sake and as evidence of our credibility, we are constantly making writerly decisions about who gets to say what and how often in the text, and who the narrator talks about, how and how often.”

Over the past at least four decades, qualitative scholars have answered the above question (whose voice?) in various ways by employing techniques such as analytic-descriptions, narrative unfolding, and sociobiography (Warren and Karner, 2010). Following the principles stated by these scholars, we adopt a popular approach to writing qualitative research, i.e., analytic-descriptions. With this approach, the main theme and its subthemes in this study were developed from ethnographic and in-depth interview data. Two rhetorical devices are typically used in the analytic-descriptive approach to representing the data: (i) the typology or the continuum, a kindred idea of what anthropologist Clifford Geertz (1988, p. 78) described as “I-witnessing”; and (ii) placing the people studied at the centre of the analysis (Warren and Karner, 2010). Given the nature, scope and purpose of this study, the second device (placing people at the centre) was adopted in the analytic-descriptions. Each data point (i.e., experiences of each informant) were analysed and connected within a coherent social context, towards arriving at a collective story. The weaving of individual voices together to tell a collective story, scholars argue, can be a powerful mode of representation (see for example Duneier, 1999).

While the broader collective story draws on a set of 30 in-depth interviews, this article employs Shalini’s story as an in-depth representative case, a common qualitative strategy used to analytically elaborate and demonstrate how the framework operates empirically, as discussed above. Shalini’s experiences are used as illustrative of the key dimensions of intimate boundary making towards arriving at the collective story of the informants.

#### Data collection and analysis

Data collection involved 30 in-depth interviews conducted between early 2023 to 2024 across Bengaluru, Mumbai, Pune [see Table 1 for Demographic overview of the interview participants (*N* = 30)]. The interview protocol was adapted iteratively over the course of fieldwork. As data collection progressed, emerging themes that had not been anticipated in the initial protocol informed revisions to subsequent interview questions, reflecting a responsive and evolving relationship between data collection and analysis. The semi-structured nature of the interviews allowed for open, in-depth discussions, ensuring a comprehensive understanding of participants’ experiences. Interview discussions explored participant’s experiences of dating, family expectations, mobility trajectories, educational and employment transitions, and digital dating practices, to name a few. Illustrative questions included: “How have digital technologies influenced your relationship practices?”; *What has your experience with dating apps been like?”; and* “What do you typically look for in a prospective partner?” Reflexive memo-writing was maintained throughout the process. Interviews ranged from 1 to 2 h, with some extending beyond; several participants remained in contact after the formal interview, providing further contextual updates that informed the analysis.

**Table 1:** Demographic overview of the interview participants (*N* = 30).

Category	Overview
Age range	25–34 years
Gender identity	Women (17); Men (11); Non-binary (2)
Sexual orientation	Heterosexual (19); Gay (5); Queer (Bisexual/Pansexual/Biromantic) (6)
Caste	Upper caste(17); OBC(7); caste not applicable(3); caste not disclosed(3)
Religion	Hindu (25); Christian (3); Muslim(1); Mixed(1)
City of residence	Bengaluru (9); Mumbai (6); Pune (15)
Education	Undergraduate (9); Postgraduate (21)
Marital Status	Unmarried (23); Married (7)
Relationship Form *(refers to current relationship and may cut across marital categories)*	Single (10); Partnered Monogamous (13); Partnered Non-monogamous(7)
Living arrangement *(excludes married participants and single participants)*	Living in (7); LAT (same city) (3); Long distance (4)

Caste categories are analytically grouped for ease of representation and is based on participant’s self-identification. Relationship Form distinguishes monogamous, non-monogamous, partnered, single arrangements. Marital Status is operationalised based on legal status at the time of the interview; for instance, socially separated but legally married participants are categorised as married. Living Arrangements refers to whether the intimate partners are cohabiting, have a long distance relationship or are living apart within the same city.

Interview transcripts were analysed using a qualitative data analysis software, MaxQDA, and followed coding and analysis premised on the principles of Grounded Theory, particularly Constructivist Grounded Theory. The process included an initial round of memoing, theme identification, examining word frequencies and key words in contexts to closely engage with participant’s narratives and experiences. This was followed by formal coding through which the recurrent and most significant codes were synthesised into analytical themes. Coding, or the process of applying conceptual patterns systematically to raw data, is a dynamic process that Strauss and Corbin (1998) term “conceptual ordering.” Majority of the analysis through coding depends on the identification of themes and patterns within the data (Coffee and Atkinson, 1996). According to Coffee and Atkinson, coding is “…condensing the bulk of the data sets into analyzable units by creating categories with and from the data” (1996, p. 26). However, it may be emphasized that coding is a *process* of data analysis, not the analysis *itself*. Similar to theme identification, in this research, coding helped in linking concepts or instances within the data. Some of the major core codes that emerged from the dataset included *Family relationships and expectations* (e.g., delayed family disclosure, coming out to family); *Digital dating practices* (e.g., dating apps, matrimonial platforms, and social media); and *Relationship experiences* (e.g., gender dynamics in dating, the role of friendship, non-normative relationship arrangements). These codes were progressively compared and grouped into broader analytical themes. The analysis with and via coding was reflexive and iterative, moving between empirical data from the interviews, and conceptual interpretations which informed the development of a novel analytical framework which we discuss below.

Figure 1 illustrates a Code Relations Browser which demonstrates broad co-occurrence patterns across selected analytical themes. Here each square represents the co-occurrence of two codes within the dataset; larger and more intensely coloured squares indicate greater overlap between codes. Boundary work consistently co-occurs with gender/sexuality, geographic contexts, family relations, qualities sought in a partner, and relationship experiences (such as break up, opting for open relationships etc.). This pattern suggests that negotiations of intimacies are shaped through the intersection of geographic, social, digital alongside familial expectations, past relationships experiences and future oriented aspirations. It may be noted here that Figure 1 is an analytical overview rather than a quantitative measure of association between codes.

**Figure 1:**
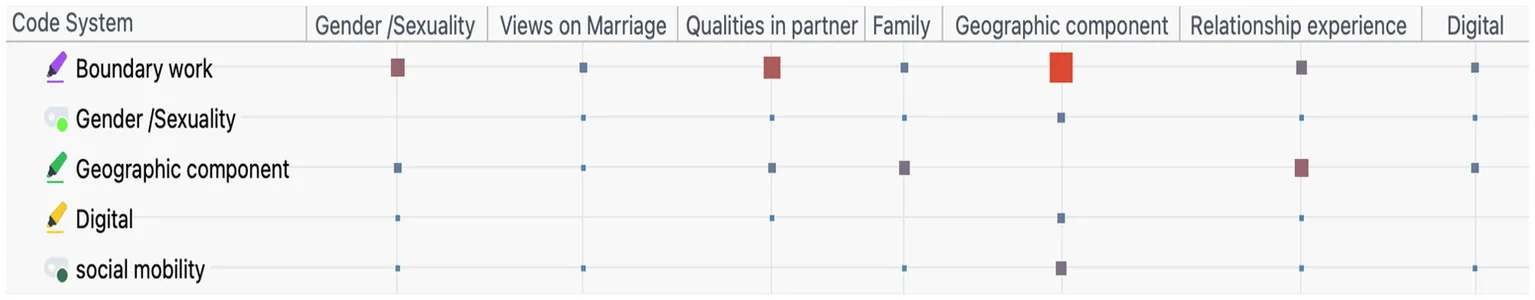
Code relations across key analytical themes in 30 interviews (MaxQDA).

In the process of formal coding, data were compared to uncover their patterns and also the *variation* in their patterns. As explained above, the analytical process has been inherently comparative, since Grounded Theory overarchingly encourages researchers to detect and show the range of variation in the concepts that emerge in the course of the analysis. Data were examined on the basis of existing concepts and their properties in order to detect patterns and thus expand the categories’ conceptual range. This process of iterative coding enabled the development of a novel theoretical framework, *Im/Mobile Boundaries of Intimacies*, through which we are able to examine the *simultaneity of culturally specific intimacies*, an aspect that neither the New Mobilities Paradigm nor the Boundary Work framework could capture fully alone. This *simultaneity* enabled by the analytical framework can be examined in respect of negotiations and constructions of intimacies across multiple geo-social scales and varied social locations, as illustrated in the following section.

#### Im/Mobile Boundaries of Intimacies: building a theoretical framework

Contemporary boundaries are dynamically shaped, negotiated and experienced through a complex interplay of geographic, social and digital factors. In the novel framework, these three factors (geographic, social, and digital) become the three fundamental pillars: (1) Geographic Location; (2) Social Mobilities; and (3) Access to Digital Spaces, on which we pivot the understanding of “Im/Mobile Boundaries of Intimacies”, that is, how people navigate boundaries of intimacies.

##### Geographic location

This pillar provides a foundation to examine spatial dimensions and the ways in which people engage across diverse geographical scales, spanning local contexts to international migration and mobilities. This includes voluntary geographical movements which refer to the choices people make to relocate from one place to another. The reasons for relocation are often nuanced and may be attributed to reasons of work, education, or personal reasons and its potential for access to and accumulation of fungible socio-cultural or economic capital (Bourdieu, 1986). Our understanding and functioning of geographic location as a pillar is inspired by another cultural anthropological framework, Gendered Geographies of Power (or, GGP), developed by cultural anthropologists (Mahler and Pessar, 2001). GGP, in short, analyzes gendered social agency, particularly in the context of migration, transnationally: “Gendered geographies of power” is a framework for analysing people’s gendered social agency - corporal and cognitive - given their own initiative as well as their positioning within multiple hierarchies of power operative within and across many terrains” (Mahler and Pessar, 2001, p. 447). We consciously choose to include a geographic aspect into our proposed framework since we believe this amplifies the nuances of not only geographic mobilities and complex power networks, but also *of social mobilities* simultaneously, particularly in cross-contextual comparative analyses such as the ones we offer in this research (Chaudhuri et al., 2014).

##### Social mobilities

Our inspiration for the second pillar of the framework, Social Mobilities, is derived from Bourdieu’s classical work on the forms of capital, particularly focusing on social capital and the idea of ‘soft’ power (Bourdieu, 1986; Bourdieu, 2000). According to Bourdieu (2000), social mobility is a function of interplay of economic, cultural, and social capital. It is shaped by the shifts in cultural and social positioning of an individual within given contexts. In popular parlance and public imagination, negotiations of intimacies are still embedded within the universal social imaginaries of marriage and the family, at least for the geographic context in this research (India, and by extension, South Asia). In a marriage-prioritised society such as in India, marrying into a “better” family—one that is propertied, well-educated, and endogamous—can significantly impact a person’s social mobility, associating their social standing closely with their marital status. Whether a person is married or not shapes their social positioning; however, this is complicated further with qualitative axes of differences or the person’s intersectional position, argued by scholars as intersectional im/mobilities (Chaudhuri et al., 2024; Shukla and Chaudhuri, 2023). Being unmarried affects women’s social positions disproportionately far more than men’s, or further, whether the woman is employed or educated, living with a disability complicates and shapes her mobility.

In this research, social mobility is also intricately linked to amorphous boundaries that typically define the ‘middle class’ in India. Such boundaries often include proficiency in English, aspiration for higher education, and salaried employment resulting in increased financial independence (Jodhka and Prakash, 2016; Bhandari, 2019). These factors collectively contribute to shifts in a person’s social position, enabling them to engage in negotiations around normative intimacies structures. Furthermore, it is shaped by people’s access to social capital resources, which is determined by their network-constellations, the extent and quality of the variety of capitals owned by those connections, and the fulfilment of reciprocity expectations (Baviskar and Ray, 2011, Bhandari, 2020). According to Illouz (2007), a person’s social identity is defined by their emotional attitude and style, which play a crucial role in the acquisition of networks and the development of social capital. This, in turn, facilitates social mobilization within intimate networks, offline or online.

##### Access to digital spaces

The third pillar of our proposed framework is strongly aligned with the digital turn in contemporary urban India, and globally. This pillar, we believe, rests on the power dynamics of *access* to the digital, involving networked individuals and social publics interconnected through digital platforms (Castells, 2011; Wellman et al., 2006). The New Mobilities turn (discussed above) acknowledges the permeation of the digital in our everyday lifestyle, which sociologists Mimi Sheller and John Urry capture in the following: “Even the most intimate ‘private’ is no longer entirely ‘personal’ or ‘inner-worldly’. Where the neural networks of the brain stop and the electronic networks of information begin is unclear” (Sheller and Urry, 2003, pp. 117). Not surprisingly, contemporary contexts are digitally saturated and this requires delving into access to digital technologies, platforms, and digital literacy to traverse these platforms as a significant aspect that affects geo-social mobility (Lupton, 2014; Markham, 2013, 2016; Gajjala, 2012). Therefore, we argue that the geographic and the social need to be interwoven with the digital, and that the digital becomes our third and final pillar for this novel analytical framework.

This third component of the analytical framework is also grounded in Urry’s (2007) examination of the impact of mobile devices on spatial mobility as it delves into the multifaceted ways in which these technologies reshape how individuals navigate physical spaces. Furthermore, mobile technologies reshape the dynamics of how individuals move through and perceive the spaces around them. This ushers in a new era of connected mobility that is not just spatial but cognitive in terms of flows of ideas and information, imaginations that can be accessed through the digital. However, contemporary digital technologies possess a distinct historical trajectory, yet they echo longstanding inquiries within social science scholarship concerning race, class, gender, nationalism, sexuality and additionally, caste in the Indian context (Risam, 2015; Dattani, 2025; Philip, 2024).

Given the contentions around power dynamics that govern the digital, it is further important to underscore the non-uniform nature of digital access, emphasizing that disparities extend beyond mere technological availability (Hargittai, 2010). These disparities are rooted in economic inequalities, cultural differences, and varying infrastructural development and manifest in multifarious ways, such as hindering educational and informational mobility. Scholars such as Lambert (2016) argue that on platforms like Facebook and Instagram, users constantly negotiate social mobility and social status, but these negotiations are shaped by people’s intersections and ability to claim unique digital literacy. A growing body of research also ties the internet with the operations of acquiring social capital and negotiating social mobility.

The analytical framework of Im/Mobile Boundaries of Intimacies, comprising of the three conceptual pillars discussed above, therefore offers a novel vantage point to examine intimacies and their boundaries in cross-cultural contexts.

Furthermore, this analytical framework facilitates understanding of gendered boundary work, particularly bright and blurred (heteronormative) boundaries (Alba, 2005). Such boundaries are influenced by im/mobile positions people occupy within both digital and non-digital realms. Social im/mobilities associated with gender norms significantly shape how particularly women, queer, and trans people navigate these spaces. They make deliberate choices about where to establish strict boundaries and where to blur them. This is observed in practices such as sharing of mobile numbers, or of Instagram handles on dating platforms such as Tinder or Bumble, or deciding how much personal information to display on public dating profiles.

In the next section, we revisit Shalini’s narrative (discussed under Introduction) and illustrate the *application of the analytical framework*.

#### Revisiting Shalini’s experiences through the lens of Im/Mobile Boundaries of Intimacies

Shalini’s geographic migration to Bengaluru from her relatively familiar life in Mumbai shielded by family symbolized her quest for independence. This change of *geographic location* exposed Shalini to varied practices of dating. However, she also acknowledges a persistent challenge that change of geographic location brought for her: an inherent tendency to overthink and overanalyze. This may have emerged from her need for safety, concerns around meeting strangers, and the reality of gender-based violence prevalent in the country. Shalini is aware of this gender power asymmetry (see, for e.g., Anderson, 2023). For Shalini, geographic movement brought with it exposure to new social worlds prompting reflection on taken from granted assumptions around sexuality, intimacies, relationships, thus expanding her horizons of imagining intimacy and future relationships.

Further, Shalini’s narrative sheds light on the societal pressures and expectations surrounding dating and relationships, which directly influenced her *social mobilities.* Her trajectory reflects a significant transition from traditional, family-influenced matrimonial decisions towards a realm of personal autonomy and self-determined choices in relationships. Her progression from a state of indecision - wrestling with the idea of rejecting suitably matched partners presented by her family - to a position where her personal needs and desires take precedence encapsulates a broader shift. This change is underpinned by her physical relocation to a new city, her engagement with digital dating platforms, the expansion of her social networks, and her attainment of financial independence. Through her experiences, we observe a meticulous process of symbolic boundary drawing, wherein Shalini assesses potential partners not just on surface-level attributes but on deeper considerations of ‘worthiness’ and ‘safety’ in intimate engagements. Initially perceiving no valid reasons to dismiss a “nice” suitor recommended by her mother, Shalini gradually adopts a more discerning approach in establishing criteria for potential life partners, including considerations for intimacies. The concept of intimacies thus broadens *beyond* conventional marriage to include cohabitation before marriage. Simultaneously, the definition of a suitable partner is no longer limited to merely being a “nice guy,” incorporating nuanced symbolic attributes reflective of the individual’s evolving criteria for compatibility and fulfilment. Shalini revealed that as she advanced in her professional and intellectual life, her method of evaluating those around her - whether potential friends, romantic interests, or prospective matches in a meeting to facilitate an arranged marriage - increasingly focuses on the way an individual reasons and thinks about the world. She stated, “My constant quest to discover someone whose intellect resonates with mine, I believe, has served as a barrier, deterring me from experiencing intimacy.” While recognizing that setting these boundaries may pose a challenge in the pursuit of intimacies, Shalini maintains that such criteria are crucial for her. She evaluates these qualities through the nature of conversations she has with others, whether through messaging on dating apps such as Bumble or during in-person meetings. Through conversations, Shalini engages in performing boundary work to ascertain if a person meets her standard of decency before considering further interactions in person.

A critical aspect that surfaces within digital dating spaces is the cognitive and emotional strategies that women employ to navigate this digital landscape safely. Anderson (2023) describes this phenomenon as hermeneutic labour building on emotional labour that women disproportionately perform in the contemporary dating context (Hochschild, 1979). Shalini’s engagement with boundary work online and offline illustrate how boundary work is gendered, and speaks directly to im/mobilities related to *access to digital spaces.* Shalini shares her experiences of having been on dating apps in both Mumbai and Bengaluru. For her, one noticeable distinction lies in the variety of profiles one experiences:

“Mumbai, for me, offered a diverse mix [of choice of dating partner] - a chef, a PhD holder in finance, an IT professional, someone from HR, even a chartered accountant. It was like flipping through a catalogue of varied interests and professions. On the other hand, Bengaluru seemed to present a more specialized pool, predominantly filled with IT enthusiasts or individuals tied to start-ups. You’d find someone who, after dabbling in IT, decided to venture into the world of coffee shops.”

Whether it is the diverse, more cosmopolitan dating pool of Mumbai or the more homogenous dating pool of Bengaluru, incidents related to women’s safety concerns stood out distinctly for Shalini in her online dating experiences. Further, these experiences also illustrate how boundary work around “safe” and “desirable” connections is carried out by her. In Bengaluru, she matched with a man originally from Goa and who was younger and “genuinely sweet.” Their online connection felt safe; in fact the safest she had ever had on a dating app. But she reported:

“The moment we met, it turned into a revelation of sorts—the first time I realized I had physical preferences. Before him, appearances hadn’t played a crucial role in my perception of potential dates. I never judged people based on their looks. But with this boy, everything changed in those first five minutes. It wasn’t that he had done something wrong; it was just a visceral reaction. I found myself thinking, ‘This isn’t what I want.’”

In Mumbai, Shalini’s interaction with a potential match, Akaash, further underscored the complexities of boundaries in online dating experiences. Akaash had a notable educational background as a graduate from a top-tier Indian Institute of Management and held a lucrative employment in South Bombay. Shalini described him as “seemingly progressive and nice-looking.” Despite initial positive impressions, their interaction swiftly devolved due to Akaash’s inappropriate advances characterized by an invitation to his home under questionable pretenses. Shalini, instead, requested for meetings in public places. Following a series of chats mostly around differences in political views leading to a series of arguments, there was abrupt silence from Akaash. This prompted Shalini to unmatch with him on the app. However, after being unmatched, Akaash sought out Shalini on another social media platform to criticize her actions as rude. This made Shalini reflect on the safety concerns; she recounts feeling worried and uncomfortable:

This reaction from someone I initially had a great connection with, and had shared good vibes with was alarming. It genuinely unsettled me. To think that the same person was the one suggesting such an intimate meetup at his place…

The above scenarios illustrate several key themes relevant to contemporary intimacies and their intersections with the digital: the initial alignment of interests and values can be superficial, masked by underlying incompatibilities that surface in contexts involving deeper personal convictions or boundaries. Moving from digital to real-life interaction made personal safety and comfort crucial, both for handling inappropriate advances and for evaluating romantic compatibility and personal safety. Moreover, Shalini’s experience with Akaash reflects the potential for digital platforms to infringe on personal privacy and boundaries. The meticulous scrutiny of profiles and interactions (the boundary work) can thus be understood as a manifestation of these broader societal concerns, gendered & intersectional im/mobilities, faced by women and people of LGBTQAI+ orientations when navigating online spaces (Shukla and Chaudhuri, 2023).

The above discussion is illustrative of how evolving criteria (boundaries drawn, redrawn for the intimate) is a function of the flows and movement that participants in this study traverse across digital and physical spaces alike. Further these experiences demonstrate that mobility does not operate as a linear movement from constrain to freedom. Geographic, social and the digital forces inflect one another, and are constitutive in expanding young people’s intimate possibilities. This enables in experimenting with sexual, romantic and relational arrangements beyond the heteronormative, mononormative expectations. Im/mobile boundaries thus allow us to adopt a theoretical lens for understanding contemporary social life that is increasingly dependent on network, flows, and travel as scholars have argued (Salazar, 2011, 2017; Sheller and Urry, 2016; Urry, 2007; Adey, 2010). The framework allows us to capture the granularity of contemporary experience, marked by heterogeneous, uneven, and unpredictable mobilities. These Im/Mobile Boundaries of Intimacies allow us to look at the contemporary negotiations that are in flux when studying relations as inflected by flows of information, ideas around desirability, relationship. This requires ongoing boundary work across familial, digital and geographic scales.

Beyond romantic and sexual relationships, several participants also described friendships as important sites of intimacy. The expansion of intimate possibilities is not limited to experimenting with different forms of partnerships but actively evaluates meaningful intimacies beyond the couple form and horizon of marriage, resonating with Singh’s (2022) account of friendship as a durable non-kin support system among young women in India.

This reshaping of intimacies reflects a departure from the conventional trajectory towards heterosexual marriage. Further, this indicates fluidity in conceptualizations of intimacies that diverges from static, traditional frameworks of family and marriage as only sites of intimacies. Narratives across the interviews underscore a reluctance to rush into marriage or committed relationships without a genuine compatible partner. This further contributes to participants’ prolonged singlehood status. This stance is emblematic of a broader societal shift among the new middle class, marked by consumption of goods and their values towards individual fulfilment and meaningful connections over adherence to prescriptive marital norms (Upadhya, 2009; Upadhya, 2011; Brosius, 2012; Fernandes, 2011; Jodhka and Prakash, 2016). The availability of mobility - geographic, social, and digital - apparently appears to facilitate a comprehensive boundary negotiation process in the pursuit of intimate connections.

Our data indicate that participants have increasingly distanced themselves from traditional marital norms while still harbouring aspirations for relationships that approximate the form and function of a marital union in the near future. Across the wider dataset, participants negotiated identities and intimate boundaries through interlocking experiences of geographic, social and digital im/mobilities. Im/Mobilities thus enables an examination of the ambiguous transformational potential of movement that people utilize to establish, blur, and shift boundaries in relation to intimate relationships. Additionally, our data highlight how non-heteronormative people work to simultaneously fit within and transgress the boundaries of the heteronorm (Roseneil, 2007). These boundaries, while permeable, are fostering new visions of future intimacies. In a situation when a suitable or desirable match never manifests, Shalini, like most participants in this study, spoke about surrounding herself with a different social constellation: supportive communities, friends, and family and living a fulfilling life either way. Using an analytical lens of Im/mobility thus enables us to understand these social constellations and intimate boundary work across interconnected geographic, social and digital spaces, while the Boundary Work framework reveals how these mobilities shape intimate possibilities.

## Conclusion and future directions

Fluid world boundaries are constantly inflected by the uneven heterogeneous mobilities that people traverse. In Shalini’s case, as well as for others who were interviewed for this study, we observe that movements across geographical, social, and digital spaces enhance the ambition and agency of individuals. This enables them to engage in extensive boundary work and resist settling for what they describe as “something less than what they deserve.” Furthermore, accessing digital platforms and migrating for higher education and employment are some examples of such fluxes that possess the capacity to transform the ways in which young people navigate their boundaries of intimacies. In this article, we have illustrated nuanced ways in which these frameworks intersect. Further, we have examined young adults’ efforts to forge meaningful intimacies in the face of societal expectations regarding gendered/heterosexual marriage and reproduction.

Our analysis is evidence that boundary negotiations within interpersonal dynamics such as forging intimate relationships, prospective marriage, and gender and sexual identities are reshaped across geographical, digital, and social dimensions. These negotiations reflect and respond to shifts in urban accessibility, cultural norms, leisure, and consumption patterns, alongside evolving aspirations. The im/mobility experiences of Shalini and others like her narrate a complex interplay of aspirations, social imaginaries, and the tangible influences of movement on personal identity formations. These factors then keep shaping and often shifting the parameters of intimacies and prospective matches.

One fundamental premise of this research has been to build upon two key areas of scholarly inquiry through the lens of gender: (i) the New Mobilities paradigm and (ii) Boundary Work, both of which have held distinct significance in historical and contemporary social science literature. Our goal here has been to advance and contribute to the scholarship that places mobility as a central metaphor in contemporary theoretical discourse, particularly in relation to socio-cultural constructs, both experienced and imagined (Salazar and Smart, 2011). To demonstrate the interplay between mobilities and socio-cultural constructs of intimacies, we have integrated the New Mobilities paradigm with the Boundary Work Framework, thereby critically examining contemporary relational dynamics. We have utilized a representative ethnographic case to advocate for and illustrate a novel theoretical framework: *Im/Mobile Boundaries of Intimacies*. We have demonstrated this illustration using an in-depth narrative from our field data on contemporary intimacies in digital urban India exploring how distinct im/mobilities shape and influence social and symbolic boundaries in navigating intimacies in both digital and non-digital contexts.

The framework of *Im/Mobile Boundaries of Intimacies* can also be applied to examine relational dynamics in non-traditional forms and in non-intimate spheres such as in employment and education, among others. Thus, this article contributes to expanding mobility paradigms and boundary works, situating movement within various contexts encountered in the field. This work enriches scholarly discourse by integrating the New Mobilities paradigm with the Boundary Work framework to analyse people’s imaginative efforts in their daily life.

In conclusion, the ethnographic insights into experiences of im/mobilities provide a compelling lens for examining contemporary intimacies in urban India. Our research highlights how mobilities, digital connectivity, and boundary negotiations transform intimate lives of young adults in contemporary urban contexts. Looking forward, the framework of *Im/Mobile Boundaries of Intimacies* has the potential to be adaptable in understanding people’s im/mobilities while performing social and symbolic boundary work in intimate relationships.

## Data Availability

The raw data supporting the conclusions of this article will be made available by the authors, without undue reservation.

## Footnotes

1 We use ‘intimacies’ to capture the plurality and fluid complexity of the negotiations that intimate relationships are built on.

2 Pseudonyms have been used throughout this article.

3 The first author, who conducted the primary data elicitation, occupies what Taylor (2011) terms as “intimate insider” position: a cisgender, heterosexual, unmarried woman of the same generational cohort as the participants, sharing similar class habitus, and formative exposure to digital culture, while researching across a wider spectrum of gender, sexual orientation, and relationship arrangements than her own lived experience. This positioning shaped both access, rapport and reflexive interviewing required to navigate interactions over the course of the fieldwork.
